# How Does Perceived Neighborhood Environment Affect Commuting Mode Choice and Commuting CO_2_ Emissions? An Empirical Study of Nanjing, China

**DOI:** 10.3390/ijerph19137649

**Published:** 2022-06-22

**Authors:** Chen Cao, Feng Zhen, Xianjin Huang

**Affiliations:** 1 School of Geography and Ocean Science, Nanjing University, Nanjing 210023, China; chencao@smail.nju.edu.cn; 2 School of Architecture and Urban Planning, Nanjing University, Nanjing 210093, China; zhenfeng@nju.edu.cn

**Keywords:** perceived neighborhood environment, commuting CO_2_ emissions, commuting mode choice, mediating effect, structural equation model, China

## Abstract

Exploring the impacts of perceived neighborhood environment on commuting behavior and travel-related CO_2_ emissions helps policymakers formulate regional low-carbon transport policies. Most studies have examined the impact of the objective measures of built environment on travel behavior and related CO_2_ emissions, and few studies have focused on perceived neighborhood environment. This study develops a structural equation model and uses data from a self-administered survey of urban full-time employees in Nanjing, China to examine the direct and indirect effects of perceived neighborhood environment on commuting mode choice and commuting CO_2_ emissions. The study shows that perceived service facilities has a significant direct effect on commuting mode and a significant indirect effect on commuting CO_2_ through the mediating effect of commuting mode choice. While socio-demographic variables such as gender have a significant direct impact on commuting mode and commuting CO_2_ emissions, they have an indirect impact on commuting mode and commuting CO_2_ emissions through the intermediate variables (such as car ownership, perceived neighborhood environment and commuting distance). The conclusions of this study show that the potential of commuting CO_2_ emissions reduction in China is enormous, and that policy interventions on commuting would help developing countries such as China achieve the goals of low-carbon transport and sustainable development.

## 1. Introduction

Cities are not only the centers of economic development and human activities, but also tremendous sources of carbon emissions [1]. In cities, transportation is identified as one of the priority sectors for decarbonization [2]. However, transportation is the most non-renewable energy-dependent sector in the world [3]. Compared to other sectors, transportation has experienced the most difficulty in achieving CO_2_ emissions reduction [4]. Clearly, reducing energy consumption and CO_2_ emission in the transportation sector contributes significantly to climate change mitigation [5]. Some of the countermeasures to achieve low-carbon cities and green transport should also include reducing CO_2_ emissions from the transportation sector [6,7]. As an important travel purpose, commuting accounts for nearly 50% of the total travel [8,9]. Discovering the reasons behind commuting mode choice and related CO_2_ emissions is vital for the construction of low-carbon cities and the formulation of sustainable transport policies and schemes in regions. Therefore, encouraging non-motorized commuting and reducing CO_2_ emissions while commuting will help developing countries such as China reduce CO_2_ emissions in urban transportation.

Unlike western developed countries, China is in the process of rapid urbanization and urban construction. High-speed economic development, urban spatial expansion and increased car ownership have resulted in an increasing proportion of employees who choose cars to commute. This shift has led to rapid growth in urban transportation demand with related energy consumption and CO_2_ emissions. In 2019, transport sector CO_2_ emissions accounted for about 10% of China’s total energy-related CO_2_ emissions [10]. Meanwhile, with the acceleration of China’s urbanization process, the urban environment has also undergone tremendous changes such as diversification of land use and suburbanization of housing. Changes in the urban built environment affect residents’ travel behavior and then generate related CO_2_ emissions [5,11]. Furthermore, changes in the objective built environment of cities affect residents’ perception of the built environment. Changes in the perception of the built environment may affect residents’ travel behavior, which further affects residents’ travel CO_2_ emissions [12,13]. Therefore, examining the impact of the perceived built environment on travel behavior and related environmental consequences is important for policymakers to promote low-carbon travel behaviors by improving the urban environment.

While scholars have been increasingly interested in combining travel behavior, travel CO_2_ emissions with urbanization [14,15], urban form [16,17,18] and land use [19,20,21] to reduce CO_2_ emissions in the transportation sector, the current literature is more focused on exploring the correlations among objective measures of the built environment, travel behavior and travel CO_2_ emission [22,23,24,25,26,27]. As for the impacts of the perceived built environment on travel behavior and travel-related CO_2_ emissions, researchers have paid limited attention [28,29,30], especially regarding the impact of perceived neighborhood environment on travel CO_2_ emissions. In fact, the perceived environment not only has the mediating effect in the impact of the built environment on travel behavior [13], but also has a direct impact on active travel behavior [31,32,33]. Currently, we know little about the impact of the perceived built environment on travel modes other than active travel.

The built environment is an important determinant of travel behavior [34]. Urban-level built environment and transportation planning are of great benefit to achieving the regional sustainable planning goals [23,35,36]. With the support of the above views, numerous studies have linked the built environment with travel behavior and travel CO_2_ emissions. Some studies have suggested that built environment factors such as land use diversity and density affect travel distance and travel mode choice, and travel distance and travel mode are closely related to transportation CO_2_ emissions [37,38,39,40,41,42]. Ding et al. examined the effects of the built environment on travel distance and energy consumption and found significant differences between commuting and non-commuting trips [24]. Ten Dam found that full-time work is associated with higher energy consumption [43]. These studies suggest that the impact of the built environment may be different for different travel purposes and types of work. Therefore, further research on commuting travel of urban full-time employees is of great significance.

Most of the above studies only have focused on the impact of objective measures of the built environment on travel behavior and related CO_2_ emissions. Research on the perceived built environment has been more focused on its association with physical activity (PA) [44,45,46,47]. Other studies have focused on the relationship between perceived environment and active travel, but most of these studies only focused on the relationship between perceived environment and children or adolescents’ active commuting to school [48,49] and adults’ active travel [31,32,50], and seldom on the impact of the perceived built environment on commuting behavior and related CO_2_ emissions of urban full-time employees. Recent studies have found that perceived high land use diversity, the existence of alternative routes, perceived cycling infrastructure, aesthetic characteristics and green space can promote pedestrian traffic and bicycle traffic [31,50]. However, we know little about the impacts of the perceived built environment on travel modes other than active travel modes [29]. A study in rural China found that perceived accessibility and preference have positive impacts on the probability of choosing to walk, and safety and neighborhood harmony have positive impacts on the frequency of motorcycle and private car trips [51]. In addition, perceptions had a mediating effect in the impact of the objective built environment on travel behavior [13]. Hong and Chen found that built environment factors such as traffic convenience and density affect perceived safety from crime, and further affect walking behavior [12].

In summary, without examining the effects of the perceived built environment on travel modes other than active travel mode and on travel-related CO_2_ emissions, the above-mentioned studies focus primarily on the impact of the objective measures of the urban built environment on travel behavior and related CO_2_ emissions. However, the existing studies seldom focus on the specialized group of urban full-time employees for whom the daily commuting behavior has an important impact on transportation CO_2_ emissions. Furthermore, most of the current studies only consider the direct effect of the built environment on travel CO_2_ emissions while ignoring the mediating effect of travel behavior. Therefore, this paper develops a structural equation model to examine the direct and indirect effects of perceived neighborhood environment on commuting mode choice and commuting CO_2_ emissions of employees in Nanjing, and to better understand the mechanism of the connection among perceived environment—travel behavior—environmental consequences. The core questions of this study are: (1) Does perceived neighborhood environment affect commuting mode choice and commuting CO_2_ emissions? (2) Does commuting mode choice have a mediating effect in the impact of perceived neighborhood environment on commuting CO_2_ emissions?

This study contributes threefold to the literature. First, we examined the impact of perceived neighborhood environment rather than objective measures of built environment on commuting mode choice and commuting CO_2_ emissions, which has received less attention in the literature. Second, our research objects are urban full-time employees with relatively fixed daily commuting behavior. Their commuting behavior has an important impact on urban transportation CO_2_ emissions as research has found that full-time work is associated with higher energy consumption [43]. Third, we used a structural equation model to examine the mediating effect of commuting mode choice in the impact of perceived neighborhood environment on commuting CO_2_ emissions. Our research provides implications for the formulation of urban commuting CO_2_ emissions reduction policies.

The remainder of this paper is organized as follows. Section 2 describes the study area, self-administered survey, variables in the model and modeling approaches. Section 3 presents calculation results of commuting CO_2_ emissions and results of the structural equation model. Section 4 presents research conclusions and discussion.

## 2. Methodology

### 2.1. Study Area and Data Collection

#### 2.1.1. Study Area

This study uses Nanjing, a core city in the Yangtze River Delta region in eastern China, as the study area. Nanjing is the capital city of Jiangsu province with its socio-economic development level at China’s forefront. As with the development of most Chinese large cities, Nanjing has experienced rapid urbanization and motorization since the 21st century. From 2000 to 2019, the urban construction area increased from 194 km^2^ to 972 km^2^, the urbanization rate increased from 53.41% to 83.20% [52,53] and the number of private cars increased from 27,413 to 2,111,876 [54,55]. The rapid urbanization and motorization of Nanjing has led to corresponding changes in the commuting mode of urban employees. Employees are increasingly dependent on private cars for commuting, and commuting CO_2_ emissions have also entered a stage of rapid growth. In addition, Nanjing has continuously strengthened its efforts in the construction of urban environment and transportation infrastructure in recent years. These efforts will inevitably affect the subjective perception of urban employees on the built environment, and this in turn will lead to a new impact on the commuting mode. Therefore, based on the highly representative study area of Nanjing, this is an important case for empirical research on Chinese cities.

#### 2.1.2. Data Collection

**Figure 1:**
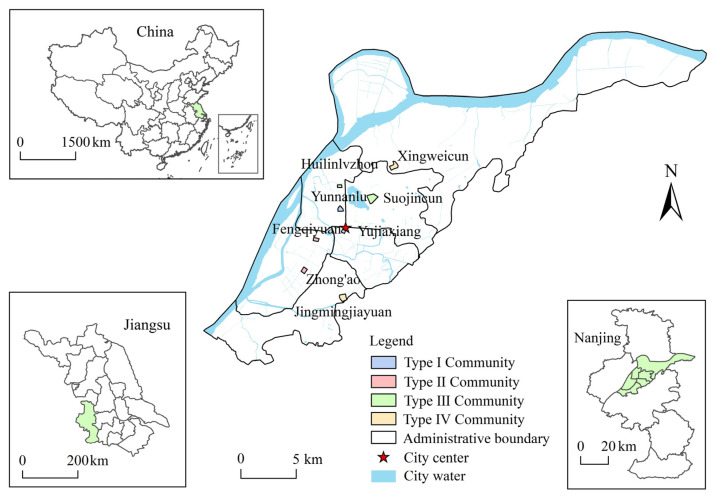
Spatial distribution of the communities surveyed in the study area of Nanjing.

Data for the study were obtained from a retrospective questionnaire survey conducted between November 2017 and January 2018. The administrative division of Nanjing includes 11 urban areas. We selected the main urban area (including 6 administrative districts) with relatively concentrated population and employment for research. Our sampling rule was to randomly select 8 administrative streets in the main urban area and randomly select a community in each street. According to the traffic environment, leisure environment and socio-demographic characteristics of the communities, we divided these communities into 4 types (Figure 1). Among the 8 communities, Yunnanlu community and Yujiaxiang community, located in the old town area, have a good traffic environment and a poor leisure environment, and belong to type I community. Zhong’ao community and Fengqiyuan community, located in Hexi new town, have a good traffic environment and a good leisure environment, and belong to type II community. Huilinlvzhou community and Suojincun community, located close to Xuanwu Lake scenic area, have a poor traffic environment and a good leisure environment, and belong to type III community. Jingmingjiayuan community and Xingweicun community, located at the edge of the main city, have a poor traffic environment and a poor leisure environment, and belong to type IV community. Before conducting the questionnaire survey, we established contact with eight community neighborhood committees to explain the purpose and use of our questionnaire and invite them to participate. After receiving affirmation, we entered the communities to issue questionnaires. The members of our research group pre-distributed the questionnaire. Based on the feedback from the research group members, we revised the questionnaires and distributed them formally.

The data were collected through face-to-face structured questionnaires filled out by random sampling method, and the respondents were recruited in community public spaces. When distributing the questionnaires, we prepared small gifts (including some daily necessities such as handkerchief papers or wet wipes, worth about USD 2) as an incentive to participants. We recorded the commuting behavior, perceived neighborhood environment and socio-demographic characteristics of full-time employees aged 18 and above. Respondents were asked to recall their commuting behavior from the past week, including the different modes of transportation they chose to travel between home and work and their corresponding time. If there was a transfer behavior, participants could record different transportation modes and their corresponding times. Respondents were also asked to provide their workplace address. Some respondents, concerned about potential privacy leakage, were unwilling to provide the address of their workplace address, and so we asked them to indicate the bus station or metro station closest to their workplace. We used the above information to calculate commuting distance and commuting CO_2_ emissions of employees. Respondents also filled in their perceptions of the neighborhood environment. These question settings used a 5-point Likert scale, with 1 representing “completely disagree” and 5 representing “completely agree”. Meanwhile, we determined whether the respondents had moved in the past five years according to the number of years they had lived in the current community to eliminate the impact of residential self-selection. Considering that employees are usually away from their home on weekdays, we chose to conduct questionnaire surveys during the weekends when employees were at home. We distributed a total of 1200 questionnaires (150 in each community), recovered 1102 questionnaires and collected 622 questionnaires from employees who had complete commuting information and had not moved in the past five years. The built environment characteristics and sample characteristics of different types of communities are shown in Table 1.

### 2.2. Variables Selection and Calculation

#### 2.2.1. Calculation of Commuting CO_2_ Emissions

Considering the availability and accuracy of data, this study uses a method commonly used internationally to calculate individual commuting CO_2_ emissions. In other words, this study uses the commuting mode and commuting distance of each employee to calculate commuting CO_2_ emissions [22,51,58,59]. The commuting distance (CD) of each sample was calculated by multiplying the average speed of each employee’s chosen mode of transportation by the corresponding commuting time. Commuting distance was calculated using the following formula:

(1) $${\mathrm{CD}}_{\mathrm{ij}}=\sum_{j=1}^{n}V_{\mathrm{ij}}{•T}_{\mathrm{ij}}$$

where ${\mathrm{CD}}_{\mathrm{ij}}$ represented the commuting distance of the employee i using the transportation mode j, $V_{\mathrm{ij}}$ was the average speed of the transportation mode j during the peak commuting period (obtained through field investigation) and $T_{\mathrm{ij}}$ was the commuting time of the employee i using the transportation mode j.

Once the commuting distance was measured for each employee using different modes of transportation, the CO_2_ emissions per commute could be calculated. According to different transportation modes, a CO_2_ emission factor was assigned to each distance. The sources of the CO_2_ emission factor values are shown in Table 2. The calculation of CO_2_ emissions was as follows:

(2) $${\mathrm{CE}}_{i}=\overset{n}{\underset{j=1}{\sum }}{(\mathrm{CD}}_{\mathrm{ij}}{•F}_{j})•2$$

where ${\mathrm{CE}}_{i}$ represented the daily commuting CO_2_ emissions of employee i, ${\mathrm{CD}}_{\mathrm{ij}}$ was the one-way commuting distance of employee i using transportation mode j and $F_{j}$ was the CO_2_ emission factor corresponding to transportation mode j. It is assumed that employees commute to and from work twice a day and use the same means of transportation each time. Due to the limitation of data acquisition, the calculation of commuting CO_2_ emissions in this study did not consider the impact of fuel type, vehicle type, vehicle speed and other factors.

#### 2.2.2. Classification of Commuting Modes

In this study, commuting mode (CM) was classified into four categories according to the CO_2_ emission factors for different transportation modes. The CO_2_ emission factors for walking and biking were both 0, and so they were classified into one category of commuting mode (namely walking/biking commuting mode). Electric bicycle, as a more common mode of motorized transportation for short-distance travel in China, had a small CO_2_ emission factor, and so it was classified as the electric bicycle commuting mode. The CO_2_ emission factors for public transport such as subway, bus and unit shuttle bus were relatively large, and so they were regarded as the public transportation commuting mode. The CO_2_ emission factor was the largest when private cars and taxis were chosen, and so we classified cars and taxis into one category (namely car commuting mode).

For the 622 samples collected in this paper, in terms of commuting mode choice, the proportion of employees who chose public transport commuting mode was the largest at 36.66%, followed by the walking/bicycle commuting mode for 30.23% of the employees. The proportion of employees choosing car commuting mode and electric vehicle commuting mode was 22.51% and 10.61%, respectively. Descriptive statistics of commuting modes are shown in Table 3.

#### 2.2.3. Factor Analysis of Perceived Neighborhood Environment

SPSS 20.0 was used to test the reliability and validity of the perceived neighborhood environment data in the questionnaire. The overall Cronbach’s alpha value of the data was 0.676, and Cronbach’s alpha value based on standardized items was 0.695. This shows that the internal consistency among the question items on the perceived neighborhood environment in the questionnaire reached the minimum acceptable value. At the same time, most of Cronbach’s alpha if the item deleted values of the perceived neighborhood environment items in the questionnaire did not reach 0.695 (Table 4). This indicates that the validity of the data in the questionnaire was good.

Next, we performed exploratory factor analysis on 18 variables of perceived neighborhood environment in the questionnaire. To analyze whether the perceived neighborhood environment variables satisfy the prerequisites of factor analysis, that is, whether there was a strong correlation among the items, KMO and Bartlett tests were conducted on the 18 variables of perceived neighborhood environment. It was verified that the KMO value was 0.777, and the significance of the Bartlett sphericity test value was 0.000. This indicates that the correlation coefficients among the items were both significant and suitable for factor analysis.

The factors were further rotated orthogonally using the maximum variance method to make them more convincing and explanatory. The cumulative variance contribution rate of the five common factors was 58.22%. From the rotation component matrix table (Table 5), combined with the meaning of each item, the five common factors were defined as service facilities perception, environmental quality perception, road condition perception, traffic safety perception and community safety perception, forming the latent variables of perceived neighborhood environment in the structural equation model. The means, standard deviations and standard errors of the latent variables of perceived neighborhood environment are shown in Table 6.

#### 2.2.4. Socio-Demographic Characteristics

Socio-demographic characteristics are crucial to understanding travel behavior and travel CO_2_ emissions. The existing relevant literature has proven that gender, age, income, education, occupation, household size and hukou not only affected travel behaviors [62,63], but also affected travel CO_2_ emissions [5,11,22,24,60]. In this study, the above socio-demographic characteristics were considered as exogenous variables introduced into the model. The impact of car ownership is more complicated, with some scholars believing that car ownership has a mediating effect between the exogenous variables and other endogenous variables [11,64,65]. Therefore, car ownership was set as an endogenous variable in this research. Table 7 presents the socio-demographic characteristics of the sample.

### 2.3. Structural Equation Model and Conceptual Framework

In recent years, scholars have often used structural equation modeling to analyze the complex relationships among built environment, travel behavior and related CO_2_ emissions [11,17,64,66]. Structural equation modeling is a multivariate data analysis tool that analyzes the relationships among variables based on the covariance matrix of variables. It integrates factor analysis and path analysis. Structural equation models can not only solve the endogeneity problem among variables, but also allow the existence of mediating variables [26,66]. Because there were mediating variables such as commuting distance and commuting mode in this study, it would have been difficult to support the analysis using traditional multiple regression methods. Thus, we used structural equation modeling to analyze the direct and indirect impact of perceived neighborhood environment on commuting mode choice and commuting CO_2_ emissions through the effect values.

Because of the subjective aspect of human behavior and their different life experiences, attitudinal preferences and socio-demographic characteristics, even in the face of the same urban built environment, different people have different subjective perceptions of the built environment, and their commuting mode choices and commuting CO_2_ emissions will differ to some extent. Therefore, the mechanism behind commuting mode choice and commuting CO_2_ emissions cannot be fully explained from the perspective of the objective measures of the urban built environment. The impact of perceived neighborhood environment must also be considered. In addition, commuting distance has been considered an important factor for commuting mode choice and travel CO_2_ emission [11,67]. Thus, commuting distance is included in the model in this paper. Socio-demographic characteristics significantly affect commuting mode choice and commuting CO_2_ emissions, so they are also included in the model.

**Figure 2:**
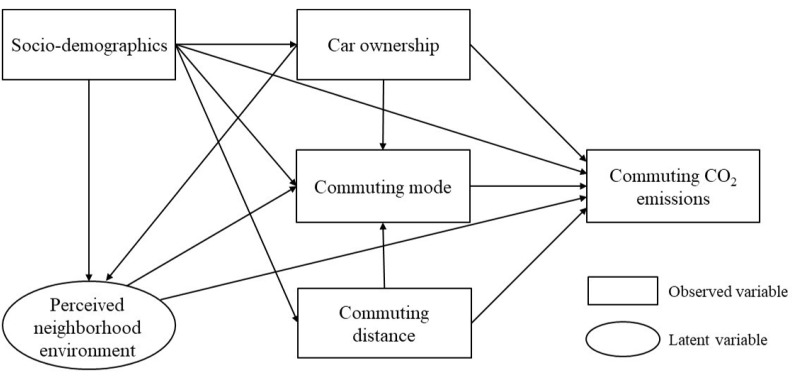
Conceptual framework for the structural equation model.

In summary, the conceptual framework of the structural equation model is shown in Figure 2. Through this conceptual framework, we can intuitively see the direct effect of perceived neighborhood environment on commuting mode and commuting CO_2_ emissions, and how perceived neighborhood environment ultimately affects commuting CO_2_ emissions through the mediating effect of commuting mode.

## 3. Results

### 3.1. Calculation Results of Commuting CO_2_ Emissions

Through calculation, the average one-way commuting distance of urban employees in Nanjing is 12.42 km, the one-way commuting time is 29.59 min, the daily commuting CO_2_ emissions per capita is 1.14 kg and the corresponding standard deviations are 13.17 km, 22.19 min and 1.82 kg, respectively. From comparison with other studies in Table 8, we found that the daily commuting CO_2_ emissions of our sample are close to the results of other scholars’ research on China [5,60], but much lower than the result of Ohnmacht et al. for Switzerland [58].

### 3.2. Goodness of Fit for Structural Equation Model

In this paper, we used AMOS 22 to build the initial model. The model was estimated using the Bollen–Stine Bootstrap because our data were not normally distributed [11,32]. We removed non-statistically significant links (*p* > 0.1) and re-estimated the model. We then modified the model according to the modification indices (MI) to obtain the final model. The model fit indices and its corresponding reference values [69] are given in Table 9. All indices show that the model fits well and is statistically significant.

### 3.3. Effects among Endogenous Variables

The relationships among endogenous variables are shown in Table 10. In terms of the direct effects among endogenous variables, the service facilities perception in the perceived neighborhood environment variables has a significant direct effect on commuting mode, which indicates that employees with a positive perception of service facilities around the community have a higher probability of choosing walking/bicycle commuting methods. It is understandable that employees have a positive perception of service facilities around the community, meaning they may live closer to the center of the main city rather than the edge of the main city, so they are closer to the workplace and are more likely to choose the walk/bicycle commuting mode. Meanwhile, car ownership and commuting distance have a significant direct effect on commuting mode. This shows that employees with more car ownership and longer commuting distance have a higher probability of choosing car commuting mode. In addition, commuting mode, commuting distance and car ownership have a significant direct effect on commuting CO_2_ emissions, which means that employees who choose to commute by car, commute longer distances or own more cars and emit more CO_2_ when commuting.

However, perceived neighborhood environment variables have no direct effect on commuting CO_2_ emissions. Service facilities perception and commuting distance indirectly affect commuting CO_2_ emissions through the mediating effect of commuting mode. That is, employees with a positive perception of service facilities around the community have a higher probability of choosing walking/biking commuting mode, and CO_2_ emissions are lower when they choose walking/biking commuting mode; employees with longer commuting distance are more likely to choose the car commuting mode, and CO_2_ emissions are higher when choosing the car commuting mode. Car ownership indirectly affects commuting mode choice through the mediating effect of service facilities perception. In addition, car ownership indirectly affects commuting CO_2_ emissions through the mediating effect of service facilities perception and commuting mode.

### 3.4. Effects of Socio-Demographic Variables on Endogenous Variables

The relationships between socio-demographic variables and endogenous variables are shown in Table 11.

Among socio-demographic variables, only income directly impacts on car ownership. This indicates that high-income employees own more cars.

Socio-demographic variables directly impact perceived neighborhood environment. Male employees have a positive perception of road conditions and a negative perception of environmental quality, which means that male employees better understood road conditions, while female employees better understood environmental quality. Older employees have a positive perception of traffic safety. Freelance employees have a negative perception of traffic safety and a positive perception of road conditions. Meanwhile, employees with local hukou have a positive perception of environmental quality. In addition, socio-demographic variables also have significant indirect effects on perceived neighborhood environment, which comes from the mediating effect of car ownership. Higher-income employees own more cars, while employees with more cars have a positive perception of community safety and a negative perception of service facilities. It is not difficult to understand that most of the high-income employees live in high-end communities, and the safety of such communities is more guaranteed; employees with more cars can easily reach farther distances to obtain services, so they have a negative perception of service facilities around their communities.

Socio-demographic variables directly impact commuting distance, commuting mode and commuting CO_2_ emissions. Male employees, employees with larger household size and employees with local hukou commute longer distance, while freelance employees commute relatively shorter distances. Male employees, highly educated employees and employees with local hukou have a higher probability of choosing the car commuting mode, while older employees and freelance employees have a higher probability of choosing the walking/biking commuting mode. Meanwhile, male employees and freelance employees emit more CO_2_ when commuting. In addition, socio-demographic variables also indirectly impact commuting mode and commuting CO_2_ emissions. This comes from the mediating effects of car ownership and commuting distance. Higher-income employees own more cars, and so they have a higher probability of commuting by car and emit more CO_2_. Male employees, local hukou employees and employees with larger household size have longer commuting distances, and so they are more likely to choose cars to commute and emit more commuting CO_2_ emissions. Freelance employees have relatively shorter commuting distances, and so they have a higher probability of choosing the walking/bicycle commuting mode; this results in lower commuting CO_2_ emissions. Highly educated employees are more likely to choose cars to commute, and so their commuting CO_2_ emissions are relatively higher. Older employees are more likely to choose the walking/biking commuting mode, and so their commuting CO_2_ emissions are relatively lower.

In general, the indirect effects of socio-demographic characteristics on commuting CO_2_ emissions are more significant than the direct effects, and so we cannot ignore the mediating effects of commuting behavior, including commuting distance and commuting mode.

## 4. Conclusions and Discussion

Taking Nanjing as the case and using questionnaire data to estimate the conceptual model of a structural equation model, this study examined the direct and indirect effects of the perceived neighborhood environment on commuting mode choice and commuting CO_2_ emissions of urban full-time employees. The following main conclusions and policy implications were determined:

(1) Using full-time employees in Nanjing as a sample, the average daily commuting CO_2_ emission per employee is 1.14 kg. If Chinese people work 250 days per year, each urban employee would emit 285 kg of CO_2_ every year due to commuting. It is evident that commuting is an important part of residents’ daily travel, and its CO_2_ emission reduction potential is enormous. If we can encourage employees to shift from high-carbon car commuting to green and low-carbon walking/biking and public transportation commuting from the perspective of changing their commuting behavior, it will not only alleviate traffic congestion in Chinese large cities and promote the construction of low-carbon cities in China, but also take part in achieving China’s carbon neutrality target by 2060.
(2) Among the perceived neighborhood environment variables, the service facilities perception directly affects commuting mode choice, and perceived neighborhood environment ultimately affects the commuting CO_2_ emissions of employees indirectly through the mediating effect of commuting mode. Therefore, in climate change mitigation, it is more beneficial to change residents’ behavior patterns through urban planning tools. In some developed countries, perceived safety and aesthetic characteristics often promote walking and cycling [30,50], so improving residents’ perceptions of safety and aesthetics can promote low-carbon travel. In developing countries such as China, a good service facilities perception may be more important for promoting low-carbon commuting mode choice and reducing related CO_2_ emissions. Therefore, improving service facilities around communities should become one of the key dimensions of urban low-carbon transportation construction in China.
(3) Commuting distance and commuting mode directly affect commuting CO_2_ emissions, and commuting distance indirectly affects commuting CO_2_ emissions of employees through the mediating effect of commuting mode choice. Since the impact of perceived neighborhood environment on commuting mode choice is limited (only the impact of service facilities perception is significant), shortening the commuting distance of employees and promoting their choice of walking/bicycle commuting are some of the effective measures to reduce the commuting CO_2_ emissions. In the past two decades, China’s urban form has continued to expand, and the job-housing imbalance is one reason for the increasing commuting distance of urban employees [70,71]. Thus, it is necessary to develop compact urban forms in the future. Meanwhile, with the advancement of internet communication technologies, more diverse forms of work can be explored. Current studies have also proven that coworking, working from home or teleworking could reduce energy consumption and greenhouse gas emissions [58,72]. In addition, accelerating the construction of pedestrian greenways, bicycle paths and public transportation systems, and advocating low-carbon travel behavior are of great significance for encouraging urban employees to choose a green commuting mode and reducing commuting CO_2_ emissions. China’s first urban mobility industry report, titled “The 2021 Urban Sustainable Mobility Observation Report”, showed that public transportation to car commuting time ratios in eight big cities in China were generally from 1.5 to 2.5 [73], indicating that the priority development strategy of public transportation in China still needs to be improved.
(4) Socio-demographic characteristics such as gender, occupation and car ownership directly influence commuting CO_2_ emissions and indirectly influence commuting CO_2_ emissions through mediating variables of commuting distance and commuting mode. These conclusions are beneficial to formulate commuting CO_2_ reduction policies for specific groups. For example, for groups with a high propensity to commute by cars—this paper refers to male employees, higher-income employees and employees with more car ownership and local hukou—the Chinese government should increase the cost of their car use. Through measures such as car purchase restrictions, higher vehicle purchase taxes and parking fees, the Chinese government can control the use of cars by urban employees within a reasonable range and encourage such employees to choose a lower-carbon commuting mode, thereby reducing CO_2_ emissions from commuting.

Overall, this study fills a research gap on the effects of perceived neighborhood environment on commuting mode choice and related CO_2_ emissions, providing some new evidence for the current construction of sustainable transport and low-carbon cities in China and other developing countries. The main limitation of this study is related to the use of CO_2_ emission factors, which are closely related to fuel type, vehicle type, vehicle speed and other factors. Due to the difficulty in obtaining such data, this study did not take these factors into account and could only estimate the model using the average CO_2_ emission factor for each mode of transportation.

## Figures and Tables

**Table 1:** Sample characteristics and built environment characteristics of different types of communities.

Community Type	Type I	Type II	Type III	Type IV
**Average commuting distance (km)**	11.13	12.19	14.38	12.13
**Standard deviation**	14.70	12.14	14.11	11.33
**Built environment characteristics**				
Traffic environment	good	good	poor	poor
Leisure environment	poor	good	good	poor
**Sample characteristics**				
Proportion of car ownership (%)	46.67	69.66	80.52	62.66
Proportion of personal monthly income greater than CNY 10,000 (%)	21.21	27.59	27.27	22.78
Proportion of Bachelor/College degree and above (%)	70.30	77.93	88.31	75.32
Proportion of local hukou ^1^ (%)	64.24	70.34	85.71	65.19

^1^ China’s hukou system refers to a household registration system required by law to officially identify every citizen as a resident of a certain area. Under this system every citizen is categorized according to the type of hukou (agricultural/non-agricultural) and the place of hukou registration (urban/rural areas) [56,57].

**Table 2:** CO_2_ emission factors for different modes of transportation (kg CO_2_/person·km).

Walk	Bike	Electric Bike	Metro	Bus	Shuttle Bus	Car	Taxi	Source
0	0	0.008	0.0091	0.035	0.035	0.135	0.135	Ma et al. [5]
0	0	0.008	-	0.035	-	0.126	0.126	Ao et al. [51]
0	0	0.008	0.0091	0.035	-	0.126	0.129	Yang et al. [60]
-	-	0.008	-	0.021	0.050	0.184	0.091	Lyu et al. [61]
0	0	0.008	0.0091	0.035	0.050	0.126	0.129	this research

**Table 3:** Share of each transportation mode and average CO_2_ emissions.

Variable	Lever	Sample Size	Percentage of Samples	Average Commuting CO_2_ Emissions (kg/person·day)	Standard Deviation
Commuting mode (CM)	1 = Walking/biking	188	30.23%	0	0
	2 = Electric bicycle	66	10.61%	0.1029	0.0529
	3 = Public transportation	228	36.66%	0.7059	0.6614
	4 = Car	140	22.51%	3.8657	2.0273

**Table 4:** Validity test of observed variables of perceived neighborhood environment.

Observed Variables of Perceived Neighborhood Environment	Symbols of Variables	Cronbach’s Alpha if Item Deleted
Easy and convenient walk to the nearest large supermarket or shopping mall	D1	0.644
Easy and convenient walk to the nearest bus stop	D2	0.667
Easy and convenient walk to the nearest metro station	D3	0.652
Easy and convenient walk to the nearest park or green area	D4	0.635
There are many intersections around the community	D5	0.671
There are many different roads around the community to choose from	D6	0.656
The roads around the community are in good sanitation condition	D7	0.660
The roads around the community are well illuminated at night	D8	0.656
The streets around the community are flat	D9	0.653
Most roads around the community have walking trails	D10	0.665
There are pedestrian crossing facilities around the community	D11	0.656
There are attractive natural landscapes around the community	D12	0.643
There are attractive cultural landscapes around the community	D13	0.658
There are not many fast-moving motor vehicles around the community	D14	0.690
Traffic accidents do not often occur around the community	D15	0.703
There are not many obstacles around the community (such as vehicles occupying roads)	D16	0.718
Public security around the community is very good	D17	0.645
Peace and order around the community is very good at night	D18	0.646

**Table 5:** Rotation component matrix.

Symbols of Variables	Component				
	1	2	3	4	5
	(Service)	(Environment)	(Road)	(Traffic)	(Community)
D1	0.579				
D2	0.650				
D3	0.668				
D5	0.563				
D6	0.680				
D4		0.714			
D12		0.724			
D13		0.779			
D7			0.550		
D8			0.599		
D9			0.641		
D10			0.773		
D11			0.732		
D14				0.772	
D15				0.654	
D16				0.688	
D17					0.870
D18					0.879

Note: The extraction method is principal component analysis; the rotation method is an orthogonal rotation method with Kaiser standardization.

**Table 6:** Mean, standard deviation and standard error of latent variables of perceived neighborhood environment.

Latent Variables of Perceived Neighborhood Environment	Symbols of Variables	Sample Size	Mean	Standard Deviation	Standard Error of the Mean
Service facilities perception	Service	622	3.785	0.624	0.025
Environmental quality perception	Environment	622	2.683	0.982	0.039
Road condition perception	Road	622	3.623	0.630	0.025
Traffic safety perception	Traffic	622	2.927	0.778	0.031
Community safety perception	Community	622	3.835	0.756	0.030

**Table 7:** Socio-demographic characteristics of the sample.

Variables	Lever	Sample Size	Percentage of Sample
Gender	0 = female	291	46.78
	1 = male	331	53.22
Income	1 = less than CNY 2000	30	4.82
	2 = CNY 2001–4000	115	18.49
	3 = CNY 4001–6000	134	21.54
	4 = CNY 6001–8000	96	15.43
	5 = CNY 8001–10,000	94	15.11
	6 = CNY 10,001–15,000	77	12.38
	7 = more than CNY 15,000	76	12.22
Occupation	1 = government staff	110	17.68
	2 = white collar	223	35.85
	3 = personnel in a specific technical field	115	18.49
	4 = general workers	100	16.08
	5 = freelance	74	11.90
Car ownership	1 = no car	221	35.53
	2 = own 1 car	313	50.32
	3 = own 2 or more cars	88	14.15
Age	1 = age 18–29	175	28.14
	2 = age 30–39	207	33.28
	3 = age 40–49	139	22.35
	4 = age 50–59	83	13.34
	5 = age 60 and above	18	2.89
Education	1 = junior high school and below	61	9.81
	2 = high school	77	12.38
	3 = undergraduate	386	62.06
	4 = postgraduate and above	98	15.76
Household size	1 = 1 person	68	10.93
	2 = 2 persons	123	19.77
	3 = 3 persons	265	42.60
	4 = 4 persons	85	13.67
	5 = 5 persons	67	10.77
	6 = 6 persons	14	2.25
Hukou	0 = other places	179	28.78
	1 = local	443	71.22

**Table 8:** Individual travel CO_2_ emissions in different studies.

Literature	Study Area	Time	Personal CO_2_ Emissions per Day
Ma et al. [5]	Beijing, China	2007	A work-related trip: 0.8 kg/person
Wang et al. [68]	Xi’an, China Bangalore, India	Xi’ an: 2012 Bangalore: 2011–2012	Urban transportation CO_2_ emissions: Xi’an: 0.28 kg/trip Bangalore: 0.41 kg/trip
Yang et al. [60]	Guangzhou, China	2015	Commuting CO_2_ emissions: 0.954 kg/day·person
Ohnmacht et al. [58]	Switzerland	2019	Commuting CO_2_ emissions: 3.32 kg/day·person

**Table 9:** Model fitness indices.

Statistical Test Volume	Indices Description	Criteria or Thresholds for Adaptation	Model Results
Absolute fit measurement			
χ^2^	Chi-square value	Significant probability value *p* > 0.05	*p* = 0.469
SRMR	Standardized root mean square residual	<0.05	0.0345
RMSEA	Root mean square error of approximation	<0.05	0.003
GFI	Goodness-of-fit index	>0.90	0.968
AGFI	Adjusted goodness-of-fit index	>0.90	0.954
Incremental fit measurement			
NFI	Normed fit index	>0.90	0.938
RFI	Relative fit index	>0.90	0.917
IFI	Incremental fit index	>0.90	1.000
TLI	Tacker–Lewis index	>0.90	1.000
CFI	Comparative fit index	>0.90	1.000
Parsimonious fit measurement			
PGFI	Parsimony goodness-of-fit index	>0.5	0.672
PNFI	Parsimony-adjusted NFI	>0.5	0.698
χ^2^/df	Chi-square/degree of freedom	1–3	1.004

**Table 10:** Standardized direct, indirect and total effects of endogenous variables on one another.

Variables Symbol	Effects	Service	Car Ownership	CD	CM
CM	Total effect	−0.098 ***	0.223 ***	0.440 ***	-
	Direct effect	−0.098 ***	0.209 ***	0.440 ***	-
	Indirect effect	-	0.013 **	-	-
CE	Total effect	−0.050 ***	0.283 ***	0.445 ***	0.508 ***
	Direct effect	-	0.170 ***	0.221 ***	0.508 ***
	Indirect effect	−0.050 ***	0.113 ***	0.224 ***	-
Service	Total effect	-	−0.133 ***	-	-
	Direct effect	-	−0.133 ***	-	-
	Indirect effect	-	-	-	-
Community	Total effect	-	0.135 ***	-	-
	Direct effect	-	0.135 ***	-	-
	Indirect effect	-	-	-	-

Note: The above values are all standardized values. ** and *** represent statistical significance at the 5% level and the 1% level respectively. Links that are not included in the model after re-estimation are indicated by “-”.

**Table 11:** Standardized direct, indirect and total effects of socio-demographic variables on endogenous variables.

Variables Symbol	Effects	Gender	Age	Income	Education	Occupation	Household Size	Hukou
Car ownership	Total effect	-	-	0.273 ***	-	-	-	-
	Direct effect	-	-	0.273 ***	-	-	-	-
	Indirect effect	-	-	-	-	-	-	-
Service	Total effect	-	-	−0.036 ***	-	-	-	-
	Direct effect	-	-	-	-	-	-	-
	Indirect effect	-	-	−0.036 ***	-	-	-	-
Environment	Total effect	−0.121 ***	-	-	-	-	-	0.142 ***
	Direct effect	−0.121 ***	-	-	-	-	-	0.142 ***
	Indirect effect	-	-	-	-	-	-	-
Road	Total effect	0.073 *	-	-	-	0.098 **	-	-
	Direct effect	0.073 *	-	-	-	0.098 **	-	-
	Indirect effect	-	-	-	-	-	-	-
Traffic	Total effect	-	0.200 ***	-	-	−0.117 ***	-	-
	Direct effect	-	0.200 ***	-	-	−0.117 ***	-	-
	Indirect effect	-	-	-	-	-	-	-
Community	Total effect	-	-	0.037 ***	-	-	-	-
	Direct effect	-	-	-	-	-	-	-
	Indirect effect	-	-	0.037 ***	-	-	-	-
CD	Total effect	0.106 ***	-	-	-	−0.100 **	0.077 *	0.078 *
	Direct effect	0.106 ***	-	-	-	−0.100 **	0.077 *	0.078 *
	Indirect effect	-	-	-	-	-	-	-
CM	Total effect	0.196 ***	−0.078 **	0.061 ***	0.071 *	−0.134 ***	0.034 *	0.105 ***
	Direct effect	0.149 ***	−0.078 **	-	0.071 *	−0.09 **	-	0.071 **
	Indirect effect	0.047 ***	-	0.061 ***	-	−0.044 **	0.034 *	0.035 *
CE	Total effect	0.210 ***	−0.04 **	0.077 ***	0.036 *	−0.016	0.034 *	0.071 ***
	Direct effect	0.087 ***	-	-	-	0.074 **	-	-
	Indirect effect	0.123 ***	−0.04 **	0.077 ***	0.036 *	−0.09 *	0.034 *	0.071 ***

Note: The above values are all standardized values. *, ** and *** represent statistically significant at 10% level, 5% level and 1% level respectively. Links that are not included in the model after re-estimation are indicated by “-”.

## Data Availability

Not applicable.
